# Mixed-Type Skyrmions in Symmetric Pt/Co/Pt Multilayers at Room Temperature

**DOI:** 10.3390/ma15228272

**Published:** 2022-11-21

**Authors:** Min He, Tiankuo Xu, Yang Gao, Chaoqun Hu, Jianwang Cai, Ying Zhang

**Affiliations:** 1 School of Physics and Electronic Information, Yantai University, Yantai 264005, China; 2 Beijing National Laboratory for Condensed Matter Physics, Institute of Physics, Chinese Academy of Sciences, Beijing 100190, China; 3 College of Materials Science and Opto-Electronic Technology, University of Chinese Academy of Sciences, Beijing 100049, China; 4 School of Physical Sciences, University of Chinese Academy of Sciences, Beijing 100049, China; 5 Songshan Lake Materials Laboratory, Dongguan 523808, China

**Keywords:** magnetic skyrmions, symmetric multilayers, DMI

## Abstract

We demonstrate the generation of mixed-type skyrmions (all are about 200 nm) that are primarily Bloch-type, hybrid-type, and a negligible amount of Néel-type in symmetric Pt/Co(1.55)/Pt multilayers at room temperature. The magnetic field dependence of skyrmion evolution is reversible. Brillouin light-scattering is used to quantitatively quantify the Dzyaloshinskii-Moriya interaction constant D in order to comprehend the mechanism. Interestingly, the D value is high enough to generate skyrmions in a symmetric sandwich structure. Micromagnetic simulations show that Néel-type skyrmions transform into Bloch-type skyrmions as the D value decreases. The interface-induced non-uniform D may be the cause to generate mixed-type skyrmions. This work broadens the flexibility to generate skyrmions by engineering skyrmion-based devices with nominally symmetric multilayers without the requirement of very large DMI.

## 1. Introduction

Magnetic skyrmions, which are chiral spin textures with a spatial extent of just a few tens to hundreds of nanometers, exhibit a number of nontrivial performances, including (i) topological stability, (ii), high storage density (iii) high mobility at ultralow current densities, and (iv) low power consumption, making skyrmions a promising basic information carrier for next-generation spintronic devices [1,2]. The so-called Bloch-type and Néel-type skyrmions are the two most well-known and extensively studied skyrmion spin textures. They differ in the direction in which the rotation from the “down” magnetization at the skyrmion’s core moves into the “up” magnetization at its edge. A Bloch skyrmion is not stable when we consider a Néel DMI vector, for example, because each form of skyrmion can have a highly unique description of the DMI vector and vice versa [1,3,4]. In addition to earlier skyrmion discoveries in symmetry-broken bulk chiral magnets [5,6,7,8,9,10,11], the Néel-type skyrmions in interfacial antisymmetric multilayers have unveiled its potential spintronic application due to the room temperature stability [12,13,14,15,16,17,18,19,20,21] and good compatibility with the existing spintronic device manufacturing techniques. In skyrmion-based spintronics devices, heterostructures comprised of 2D magnetic material and metal or other materials often play an important role due to the interfacial Dzyaloshinskii-Moriya interaction (DMI) they produce. The thin antisymmetric sandwich structure has been studied extensively due to its tunability through altering the constituent component and relative layer thicknesses, such as heavy metal/ferromagnetic/another heavy metal (HM1/FM/HM2) multilayers. Strong interfacial spin-orbit coupling in these systems causes a large interfacial Dzyaloshinskii–Moriya interaction (DMI), which is quantified by the DMI constant, D. It is well acknowledged that DMI favors the generation of skyrmions when the system is perpendicularly magnetized [22]. Meanwhile, a critical thermodynamic stability parameter κ can be used to present the skyrmion stability [23,24,25,26,27] with κ > 1 for thermodynamically stable skyrmions:

(1) $$\kappa =\left(\frac{\pi }{4}\right)\cdot \frac{D}{\sqrt{A\cdot K}}=\frac{D}{D_{c}},$$

Equation (1) state that a relatively larger value of D is favorable for generating thermodynamically stable skyrmions. To date, various methods have been employed to increase DMI, including increasing the number of interface [20,22,28], altering the magnetic layer’s composition [29], decreasing the thickness of magnetic materials to monolayer [30], and developing new interfacial DMI mechanisms [31,32,33,34] in the multilayers with perpendicular magnetic anisotropy (PMA) ferromagnet.

However, less research about skyrmions has been completed on multilayers with symmetric sandwich structures. Theoretical calculations show that when the symmetric Pt/Co/Pt sandwich structure is used as the repeating unit, the total effective DMI in the Pt/Co/Pt multilayers should vanish because the top and bottom interfaces produce an interfacial DMI of the same magnitude but opposite sign (since the DMI is a chiral interaction) [35]. Despite research indicating the crystallographic asymmetry between Pt/Co and Co/Pt interfaces in Pt/Co/Pt systems can give rise to non-null small DMI [36], according to Equation (1), small D is not conducive to the stability of skyrmion. Experimentally, the means of observing skyrmions mainly include Lorentz transmission electron microscopy (TEM) [9,37,38], spin-polarized scanning tunneling microscopy (SP-STM) [39,40,41], spin-polarized low-energy electron microscopy (S-PEEM) [12,17], magneto-optical Kerr effect (MOKE) [14,16] microscope and scanning transmission X-ray microscopy (STXM) [15,22], magnetic force microscopy (MFM) [42]. In this work, stable skyrmions are generated and observed in symmetric Pt/Co/Pt ultrathin multilayers by Lorentz TEM at room temperature with no need for significant large DMI, opening up a larger range of material options for room-temperature skyrmion.

## 2. Materials and Methods

The multilayers Ta(4)/[Pt(3)/Co(1.55)/Pt(3)]_4_ (thickness in nm, hereafter Pt/Co(1.55)/Pt) have symmetric sandwich structures, where the lower 4 denotes the layer repetition number, were deposited by magnetron sputtering on 10-nm-thick Si_3_N_4_ membranes windows for Lorentz TEM observation. The working argon pressure was 0.5 Pa and base pressure for the sputtering system is better than 5 × 10^−5^ Pa. On thermally oxidized Si wafers, identical companion films were produced concurrently for magnetic property measurements by vibrating sample magnetometer (VSM). Lorentz TEM (JEOL 2100F) has been used to observe the magnetic domains with a perpendicular magnetic field applied by gradually increasing the objective lens current. Brillouin light-scattering (BLS) measurements were performed to study DMI of the multilayers. The simulations carried out in the present work were completed using the micromagnetic code MuMax3 [43]. All results presented in this study were obtained at room temperature.

## 3. Results and Discussion

**Figure 1:**
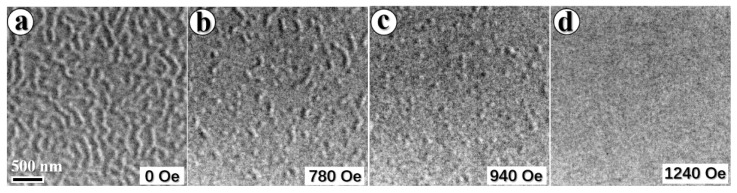
The magnetic domain evolution in symmetric Pt/Co(1.55)/Pt multilayers. Lorentz TEM Fresnel images showing the corresponding domain evolution at different magnetic fields (**a**) 0, (stripe phase) (**b**) 780, (mixed states) (**c**) 940, (skyrmions), and (**d**) 1240 Oe, (saturation state). The scale bar in (**a**) is 500 nm.

**Figure A1:**
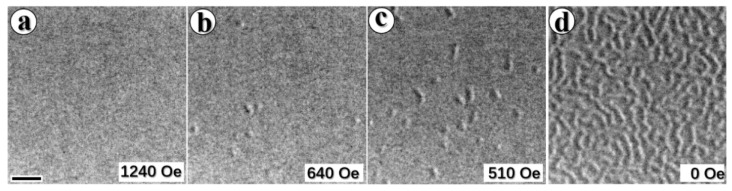
Lorentz TEM Fresnel images showing the corresponding domain evolution at decreasing the magnetic field (**a**) 1240, (saturation state) (**b**) 640, (skyrmions) (**c**) 510, (mixed states), and (**d**) 0 Oe, (stripe phase). The scale bar in (**a**) is 500 nm.

In order to describe the skyrmions in the symmetric Pt/Co(1.55)/Pt multilayers, the magnetic domain evolution under different magnetic fields is directly observed as shown in the over-focused Lorentz TEM images Figure 1. Typical demagnetized stripe phases in the Pt/Co(1.55)/Ta multilayers are shown in Figure 1a. The stripe phases become narrower with the increasing magnetic field and then skyrmion-like domains arise when the magnetic field is increased to 780 Oe, as shown in Figure 1b, and the complete skyrmion-like domains (~ 200 nm) are achieved at the magnetic field of 940 Oe (Figure 1c). In the saturated ferromagnetic phase, all skyrmion-like domains completely disappear at magnetic fields of about 1240 Oe (Figure 1d). Unlike skyrmion lattices in single crystalline bulk material, isolated skyrmions are usually observed in thin films [12,14,16,44]. One may speculate that the lack of ordering could be due to pinning associated with defects. These defects may provide a nucleation core for generating skyrmions and pinning centers for driving skyrmions [12,15]. It may also be related to the small thermodynamic stability parameter κ [28]. The domain evolution is reversible when decreasing the magnetic field (see Figure A1). This reversible evolution process indicates that the skyrmion-like domain is a thermodynamic stable state.

**Figure 2:**
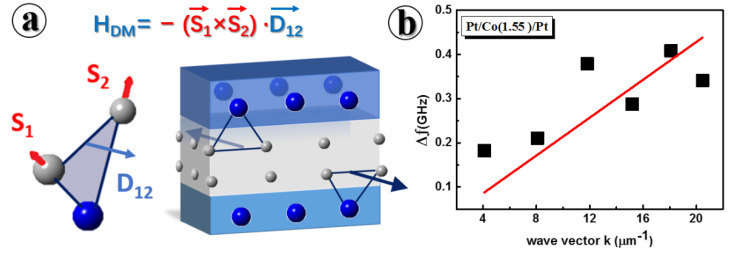
The interfacial Dzyaloshinskii–Moriya constant in symmetric Pt/Co(1.55)/Pt multilayers measured by Brillouin spectroscopy. (**a**) The schematic multilayers illustration composed of a magnetic Co layer (grey) sandwiched between two heavy metal layers of Pt (blue) with the possible DMI construction for neighbor atoms. Ideally, the total effective DMI by using the symmetric Pt/Co(t)/Pt multilayer as the repeating unit should be canceled because the top and bottom interfacial DMI exhibit the same magnitude but the opposite sign. (**b**) Frequency difference of counter-propagating spin waves as a function of wave vector in Pt/Co(1.55)/Pt multilayers, fitting well to the solid line based on Equation (2). D = 0.23 mJ/m^2^ is calculated from the slope by linear correlation. Symbols denote measured data.

The generation of a skyrmion-like domain indicates that the system’s DMI value is not zero. Figure 2a shows the DMI and membrane structure for the symmetric film. In order to quantify the interfacial DMI constant D, momentum-resolved BLS are measured by varying the incident angle. The DMI-induced frequency difference of counter-propagating spin waves is given according to the following equation:

(2) $$\Delta f\left(k\right)=\frac{f\left(-k\right)-f\left(k\right)}{2\pi }=\frac{2\gamma }{\pi M_{s}}Dk,$$

**Figure A2:**
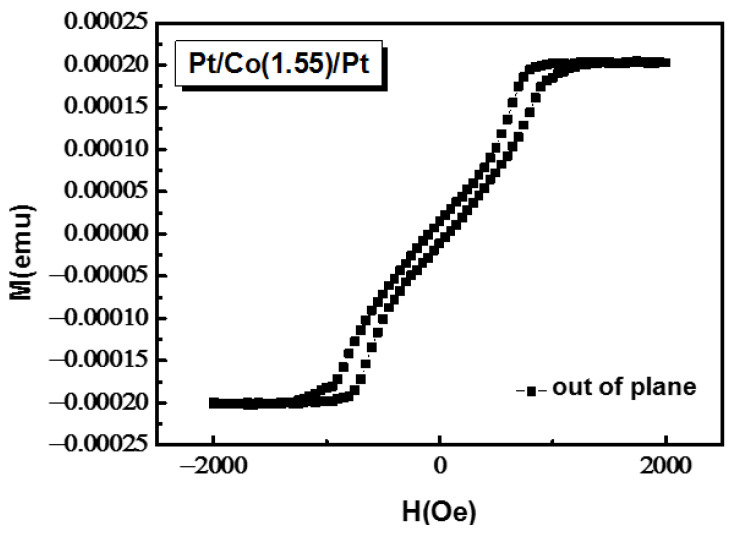
Room-temperature magnetic hysteresis loops measured along the out-of-plane direction.

It can be concluded that a linear correlation exists between $\Delta f\left(k\right)$ and k with the slope determined by D [45]. Figure 2b shows the BLS spectra for Pt/Co(1.55)/Pt, where D = 0.23 mJ/m^2^ is calculated from the slope by linear correlation when considering the gyromagnetic ratio γ=192 GHz/T [46] and $M_{s}=1320\;\mathrm{emu}/{\mathrm{cm}}^{3}$ based on VSM measurements (Figure A2). Surprisingly, moderate instead of negligible DMI is achieved in this symmetrical sample, which explains the generation of skyrmions. It should be emphasized that the DMI obtained from BLS measurements is an effective DMI averaged over the film thickness and originates from both the bottom and top interface contributions. This suggests that the DMI might originate from different roughness and intermixing effects between the bottom and top interfaces [47,48,49,50]. Considering the small size of these textures (~200 nm) and the magnitude of the DMI value, the obtained magnetic domain should be skyrmions rather than magnetic bubbles.

**Figure 3:**
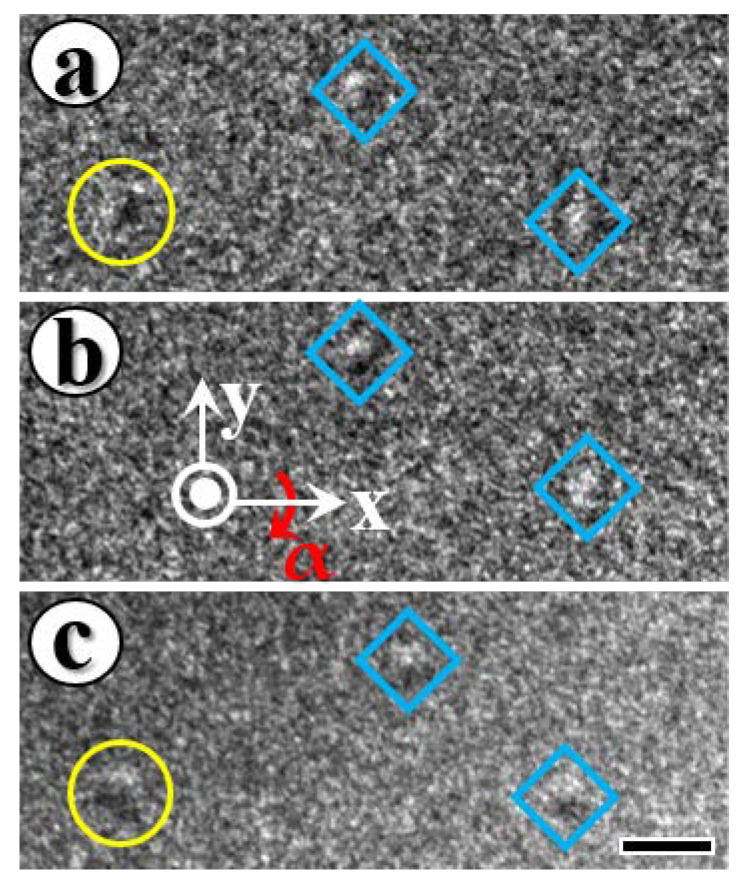
Tilting samples in a real-space Lorentz TEM at room temperature to identify skyrmion type. The tilt angle α around *x*-axis is (**a**) −18°, (**b**) 0° and (**c**) 18°, respectively. Based on the disappearance of contrast at 0° tilt and reversed contrast for opposite tilt angles, the skyrmion in the yellow circle is identified as Néel-type. The skyrmions marked with blue diamond indicate nonideal Néel-type skyrmions based on the presence of domain contrast at any tilt angle. The scale bar in (**c**) is 200 nm.

In order to confirm the spin configuration of the skyrmion texture, the sample is tilted based on the magnetic field-induced beam deflection and the contrast mechanism of Lorentz TEM. Néel-type skyrmions could be theoretically and experimentally identified by this method [44], which may be summed up as the removal of contrast at a position and reversal of contrast at approximately opposite angles. The tilting process is illustrated in Figure 3. The skyrmion marked with a yellow circle is clearly observed to have reversed bright and dark magnetic contrasts at relatively opposite tilting angles of 18° (Figure 3a,c), with no contrast at zero angle (Figure 3b), which corresponds well with the typical Néel-type skyrmion in multilayers [44]. In addition to the extremely rare Néel-type skyrmions that have been identified, the presence of domain contrast at any tilting angle indicates nonideal Néel-type skyrmions for those marked with a blue diamond.

**Figure 4:**
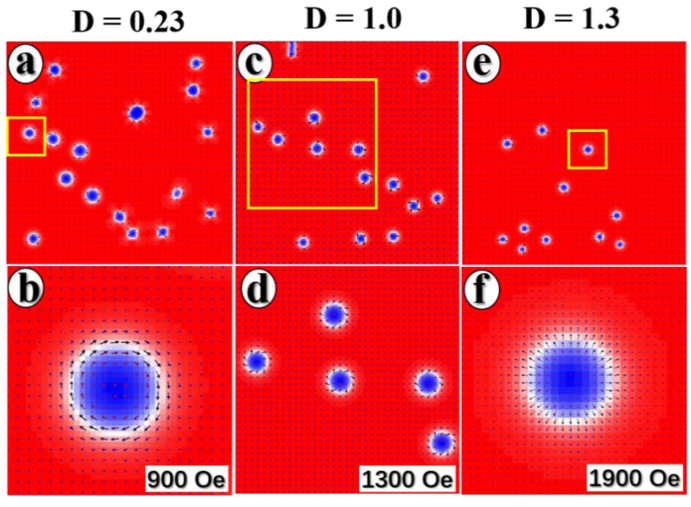
Micromagnetic simulated magnetic structure. (**a**) Bloch-type skyrmions generated at D = 0.23 mJ/m^2^, (**b**) is the enlargement of selected region. (**c**) Hybrid-type skyrmions are generated at D = 1 mJ/m^2^, (**d**) is the enlarged spin configuration of selected skyrmions. (**e**) Néel-type skyrmions generated at D = 1.3 mJ/m^2^, (**f**) represents the enlarged spin configuration of selected skyrmions.

Micromagnetic simulations were used to figure out the nonideal Néel-type skyrmion configuration. Micromagnetic simulations across a 0.6 × 0.6 μm^2^ area discretized into 2 × 2 × 2 nm^3^ cells are performed using Mumax3 software [43] based on the Landau–Lifshitz–Gilbert equation with a relax function to find the minimum energy of the system. The total free energy term can be written as $\varepsilon =\int_{V_{s}}\left(\varepsilon_{ex}+\varepsilon_{anis}+\varepsilon_{DMI}+\varepsilon_{zeeman}+\varepsilon_{dem}\right)dr$, where the exchange energy term is expressed as $\varepsilon_{ex}=A\left(\partial_{x}m^{2}+\partial_{y}m^{2}+\partial_{z}m^{2}\right)$, uniaxial anisotropy energy $\varepsilon_{anis}=K{\left(u\cdot m\right)}^{2}$, DMI energy $\varepsilon_{DMI}=Dm\cdot \left[\nabla \times m\right]$, Zeeman energy $\varepsilon_{zeeman}=-M_{s}B_{ext}m$, and demagnetization energy $\varepsilon_{dem}=-\frac{1}{2}M_{s}B_{d}m$. Here, $\;m\equiv m\left(x,y,z\right)$ represents the normalized spin at the site and u is the unit vector of uniaxial magnetic anisotropy. A, K, D, and $M_{s}$ are the exchange interaction, anisotropy interaction, DMI interaction, and saturation magnetization, respectively. $B_{d}$ is the demagnetizing field. $M_{s}=1.32\;\mathrm{kA}/m$ was chosen based on the VSM result (Figure A2). The anisotropy interaction K = 1.1 MJ·m^−3^ with u = (0, 0, 1) and the magnetic exchange stiffness A = 6 pJ·m^3^ were used here. The simulation is initialized by relaxing a state with random magnetization orientations. Then, a vertical magnetic field is applied to the initial state, and the magnetic field is gradually increased to generate skyrmions. By varying the DMI interaction, Bloch-type skyrmions are generated at D = 0.23 mJ/m^2^ when the magnetic field H = 900 Oe is applied (Figure 4a,b). Hybrid-type skyrmions are generated when D is increased to 1 mJ/m^2^ with an applied magnetic field H = 1300 Oe as shown in Figure 4c,d. Néel-type skyrmions are generated under an applied magnetic field H = 1900 Oe as shown in Figure 4e,f as D is increased until it reaches 1.3 mJ/m^2^. When all other parameters remain constant, but D is increased, the configuration of the skyrmion shifts from Bloch-type to hybrid-type and finally to Néel-type. This implies that the value of D will affect not only the size of skyrmions [28], but also the skyrmion configuration [31]. The nonideal Néel-type skyrmions configuration in Figure 3 is probably Bloch-type or hybrid skyrmions. It is worth noting that the D value of the multilayers is derived from the interface. The distribution of D values may not be uniform due to the non-uniform interface, which leads to experimentally observed mixed-type skyrmions. The tilting experiment and micromagnetic simulations support the idea of interfacial regulation of skyrmion configuration in the same material.

## 4. Conclusions

In summary, we have demonstrated the generation of small-size skyrmions (~200 nm) in symmetric Pt/Co(1.55)/Pt multilayers at room temperature. The complete skyrmions could be generated by reversible ascending and descending magnetic fields. Interestingly, moderate instead of negligible DMI is achieved in this symmetrical sample. Additional tilting experiments and micromagnetic simulations demonstrate that the skyrmion configuration is a combination of primarily Bloch-type, hybrid-type, and a negligible amount of Néel-type. The major cause leading to the generation of mixed-type skyrmions may be the non-uniform distribution of DMI brought on by the non-uniform interface. The potential to generate skyrmions in symmetric Pt/Co/Pt multilayers with moderate DMI offers new perspectives toward more flexible possibilities for skyrmion-based spintronic applications without pursuing large DMI. In addition, the effect of DMI on spin configuration also supports the idea of interfacial regulation of skyrmion configuration in the same material.

## Data Availability

Data available upon request.

## Appendix A

The saturation magnetic moment M under a saturated magnetic field is 0.0002044 emu, as shown in Figure A2. The multilayers under test are grown on a silicon wafer measuring 5 × 5 mm^2^. The total thickness of the magnetic layer is 6.2 nm. Easy to calculate the saturation magnetization $M_{s}=1320\;\mathrm{emu}/{\mathrm{cm}}^{3}$. It could also be written as $M_{s}=1.32\;\mathrm{kA}/m$.
